# Monte Carlo Guidance for Better Imaging of Boreal Lakes in the Wavelength Region of 400–800 nm

**DOI:** 10.3390/s25041020

**Published:** 2025-02-09

**Authors:** Vinh Nguyen Du Le

**Affiliations:** Department of Physics and Astronomy, University of Alabama in Huntsville, Huntsville, AL 35899, USA; v.n.du.le@uah.edu; Tel.: +1-256-824-2487

**Keywords:** Monte Carlo, boreal lake, optical properties, absorption, scattering, image contrast, Landsat

## Abstract

Boreal lake depth, one of the most important parameters in numerical weather prediction and climate models through parametrization, helps in identifying notable environmental changes across the globe and in estimating its effect on the ecosystem in remote regions. However, there is no quantitative tool to effectively estimate lake depth from satellite images, leaving scientists to infer lake depth from extrapolation of statistics by relying on certain geological knowledge (such as those used in the Global Lake Database). The bottoms of boreal forest lakes are mainly composed of woody debris, and thus spectral imaging revealing contrast of woody debris can be used to estimate lake depth. Here, we use well-established Monte Carlo software to construct spectral images of boreal lakes that house woody debris, phytoplankton, and chlorophyll. This is accomplished by modeling the dynamic optical properties of selected boreal lakes and simulating the propagation of photons in the wavelength region of 400–800 nm. The results show that the spectral image contrast of boreal lakes is not only determined by the depth level and concentration level of phytoplankton and chlorophyll in water but is also affected by the spectral shape of background absorption, especially the contribution of pure water absorption in the total absorption of lake water.

## 1. Introduction

The quantity and area of closed-basin lakes in the northern high latitude regions (from Artic to sub-Artic) have declined significantly over the past 50 years, up to 54% and 31%, respectively [1,2,3,4,5]. This decline is strongly associated with climate warming in these regions [6,7,8,9]. Therefore, accurate quantification of total water volume and volumetric change for boreal forest lakes helps in identifying notable environmental changes across the globe and in estimating its effect on the ecosystem in remote regions [10,11,12,13,14]. Currently, lake depth is commonly evaluated by combining visual analysis of satellite images provided by different resources, such as Google Earth or Landsat [15], with statistical analysis of a large-enough grid-based regions obtained from numerous global datasets, such as the Global lake Depth Database (GLDB) [16], Global lakes and Wetlands Database (GLWD) [17], and HydroLAKES database [18]. However, estimates of lake depth within these global models is problematic because 90 million of the world’s lakes range between 0.002 and 0.01 km^2^ in size [19], and the majority of them are situated in very remote areas. Moreover, depth of lakes smaller than the model grid-box are treated as sub-grid in numerical weather prediction, often leading to inaccurate estimates of small lake volumes.

To improve and optimize boreal lake depth calculation from global databases and satellite images, this paper quantitively evaluates the effects of satellite wavelengths on image contrast of woody debris within boreal lakes. Ultimately, the paper investigates the effect of boreal lake water optical properties (scattering and absorption) across the wavelength region of 400–900 nm on the reflectance image contrast and depth of penetration (location of woody debris), allowing more robust calculation of lake depth from global databases and satellite images. This is accomplished by using graphics processing unit-based Monte Carlo software to simulate the propagation of photons in different types of boreal lakes in the Finland region, where their optical properties are well-established [20]. Using the conventional cut-off at 10% for image contrast, recommendations for optimal wavelengths to image lakes of different water conditions are made.

## 2. Materials and Methods

### 2.1. Monte Carlo Model

Monte Carlo (MC) methods have been considered the gold standard to solve the radiative transfer equation (RTE) and to validate approximations for RTE solutions [21,22,23,24,25,26]. In addition to the ability to describe the propagation of photons in turbid media [27,28,29,30,31], MC can produce accurate solutions for RTE when simulating heterogenous media with high absorption or low scattering, where the diffusion approximation becomes invalid [32]. The main differences among different MC methods may include the following: ability to simulate complicated geometries in three dimensions (3-D), availability (open-source software), cost-effectiveness, computational speed, and application variety [33,34,35,36,37,38]. Over the last 20 years, the graphics processing unit (GPU)-based Monte Carlo method has been widely used to simulate photon migration in complex biological media to meet all of these criteria [39,40,41,42,43,44,45,46,47]. Furthermore, though different Monte Carlo methods have been used to support statistical analysis for dynamic changes of boreal lakes [38,48,49,50,51,52,53], direct simulations of the interaction of photons with optically active substances in lake water to obtain spectral reflectance images of boreal lakes remain scarce or yet to exist. Here, we will use MCX software to simulate photon propagation in boreal lake water. The simulation will launch 10 billion photons into a lake water volume of 5 × 15 × 3 m^3^ (X-Y-Z). Each voxel dimension is 1 cm.

**Figure 1:**
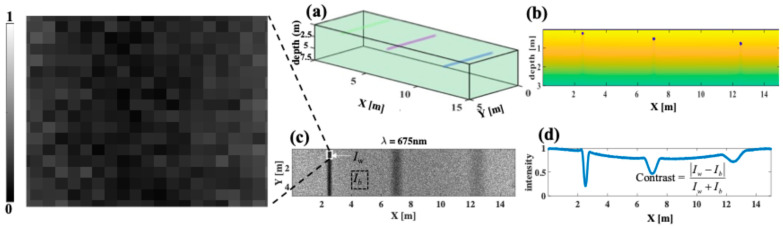
Monte Carlo simulation of photon interaction with boreal lake water and escape to the surface: (**a**) 3-D MCX model of woody debris in a simulated volume of lake water, (**b**) photon depth fluence in log-scale, (**c**) spectral reflectance image, (**d**) and the corresponding intensity profile across the X-dimension and how image contrast is calculated. Wood cylinders are located at depths of 2, 5, and 7.5 m and have a diameter of 20 cm.

Each simulation launches 1 billon photons using a gaussian beam of radius 15 m on the lake surface. The simulated surface width (Y-dimension) is within the limit of ground sampling distance of Landsat’s Operational Land Imager 2 (30 m). Each simulation takes less than 5 min to run on a computer with a standardNVIDIA GPU (NVIDIA^®^ RTX™ A6000, 48 GB GDDR6, 4 DP, 7960T). Straight cylinders along the Y-axis with diameters of 20 cm are used to simulate woody debris at different depths. Here, we will show examples for debris located at depths of 2, 5, and 7.5 m, and at a spatial distance of X = 2.5, 7.0, and 12.5 m, respectively (Figure 1). The selected depth values are within the mean depth of boreal lakes in the Finland study region [16,54,55], whereas the selected cylinder size is within the average diameter of woody debris found in this region [56,57,58]. Each simulation corresponds to each wavelength and includes three wood cylinders located at three depths (Figure 1a,b). In MCX, the default Henyey–Greenstein phase function is used to describe the scattering of light in turbid media. Here, photons in the wavelength region of 400–800 nm are our focus because they cover the majority of Landsat wavelengths used for coastal region inspections. Also, water absorption is low in this region, allowing better visualization of woody debris. The image contrast will be calculated from the image intensity values corresponding to the positions of woody debris and background lake water (Figure 1c,d), so that

(1) $$\mathrm{Contrast}=\left|I_{W}-I_{b}\right|/\left(I_{W}+I_{b}\right)$$

In Equation (1), *I_W_* is the reflected intensity at the woody debris location, and *I_b_* is the reflected intensity from background water. Equation (1) is commonly used to evaluate the performance of optical systems [59]. To calculate *I_W_*, an average of 20 central pixels were used along the X-dimension, allowing us to calculate the standard deviation, which is shown as error bars. Other MCX inputs include optical properties of woody debris and of lake water.

### 2.2. Optical Properties of Boreal Lake Waters and Woody Debris in the Study Area

**Figure 2:**
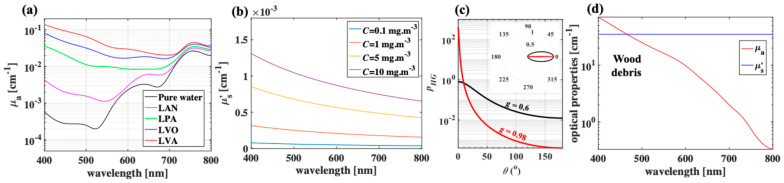
Optical properties of lake water vary significantly, depending on the abundancy of phytoplankton (major absorber) and of chlorophyll (major scatterers), and are wavelength dependent. (**a**) Absorption coefficient spectrum of pure water, lake Äntu Sinijärv (LAN), lake Päijänne (LPA), lake Vôrtsjärv (LVO), lake Valkekotinen (LVA); (**b**) lake water reduced scattering coefficient spectrum at different chlorophyll concentration *C*; (**c**) exemplary Henyey–Greenstein phase functions with normalized polar plots in inset at high and low anisotropy values; (**d**) optical properties of woody debris. Here, each lake is identified by their signature phytoplankton concentration, so that LVA represents lake water with the highest phytoplankton. Chlorophyll concentration will be varied or fixed depending on each dataset below.

In biophotonics, the absorption coefficient *μ*_a_ [cm^−1^] is commonly used to describe a medium containing many chromophores at a concentration described by a volume density and is defined as the effective absorption cross-sectional area (or size of the absorption shadow) per unit volume of the medium. Here, boreal lakes in Finland regions is selected for their well-established optical properties [20]. Specifically, the *μ*_a_ spectra of lake Äntu Sinijärv (LAN), lake Päijänne (LPA), lake Vôrtsjärv (LVO), and lake Valkekotinen (LVA) were used to represent low, intermediate-low, intermediate-high, and high levels of concentration of phytoplankton, respectively. In addition, pure water (no phytoplankton) was simulated as a baseline. The absorption coefficient of lake water at a specific wavelength can range from that of pure water to several orders of magnitude greater than that of pure water, depending on the concentration of phytoplankton. For example, the *μ*_a_ of lake Valkekotinen (LVA) water in Taivalkoski, Finland [20], is much higher than that of pure water at wavelength of 400 nm because of the dominant absorption of phytoplankton pigments (Figure 2a). The peak around 750 nm in LVA’s *μ*_a_ spectra is due to water absorption.

Similarly, the scattering coefficient *μ_s_* [cm^−1^] is commonly used to describe a medium containing scattering particles and is defined as the effective scattering cross-section (or size of the scattering shadow) per unit volume. Major scatterers in boreal lakes are chlorophyll particles [60,61]. The total scattering of lake water depends on both wavelength and concentration of chlorophyll, and can be described by Equation (2) as follows:

(2) $$\mu_{s}=\mu_{s}^{\mathrm{PW}}+\frac{550}{\lambda }{0.30C}^{0.62}$$

In Equation (2), *λ* is the wavelength in nanometers, *C* is the concentration of chlorophyll in water in mg.m^−3^, and $\mu_{s}^{\mathrm{PW}}$ is the scattering coefficient of pure water in m^−1^ [60,61]. Optical properties of pure water are extracted from Hale et al.’s study [62]. To simulate a large range of chlorophyll in water, *C* values between 0.1 and 10 mg.m^−3^ are used. Along with *μ_s_*, anisotropy factor *g* (dimensionless) is used to describe the amount of forward direction retained after a single scattering event. It is a common practice to lump *g* and *μ_s_* into the reduced scattering coefficient *μ_s_*’, where *μ_s_*’ = *μ_s_*(1 − *g*) [37,63,64,65,66]. The reduced scattering coefficient is useful to describe the diffusion of photons when there are many scattering events before an absorption event. The same practice will be used in this paper. In addition, an anisotropy factor *g* of 0.924 (forward scattering) is used for lake water [35]. In practice, *g* is computed in Equation (2) by measuring the scattering function and then utilizing the relation between *g* and the scattering phase function in Equation (3):

(3) $$g=\int_{-1}^{1}p\left(\cos\theta \right)\cos\theta d(\cos\theta )$$

(4) $$p\left(\cos\theta \right)=\left(1-g^{2}\right)/{\left[1+g^{2}-2g\;\cos\theta \right]}^{3/2}$$

In Equations (3) and (4), *θ* is the scattering angle, and *p*(cos*θ*) is the Henyey–Greenstein phase function [35]. In this paper, *p*(cos*θ*) for particles in water at a shallow depth is extracted from numerical models described by Curtis et al. [67]. Figure 2a,b summarizes the optical properties (*μ*_a_ and *μ_s_*’) of selected boreal lakes in the Finland region [20]. These lakes are selected for their well-established optical properties, while representing reasonable levels of light absorption (by phytoplankton) and scattering (by chlorophyll) in boreal lakes. Figure 2c demonstrates scattering properties by using the Henyey–Greenstein formula in both Cartesian plots and polar plots for high and low *g* values. Overall, photon absorption dominates photon scattering in lake water even at a high chlorophyll concentrations. Furthermore, because concentrations of phytoplankton and chlorophyll are varied across seasons [20], varying concentrations of these species in the simulations can reflect climatic and seasonal conditions.

Coarse woody debris is an important component of boreal forest lakes, and it affects the geometry of the lake and the retention of organic and inorganic matter [68,69,70] and is an important factor in the propagation of light in lake water. Many species of hardwood and softwood can be found at the bottom of boreal forest lakes. Here, chestnut wood (hardwood type) is selected for simulations due to its high absorption t in the visible region of the EM spectrum (Figure 2d) [71]. The reduced scattering coefficient spectrum of wood is relatively flat within λ = [400–800] nm [71], and a constant *μ_s_*’ of 35 cm^−1^ is used for degraded chestnut wood (Figure 2d) [71].

## 3. Results

### 3.1. The Effect of Phytoplankton Absorption on the Spectral Contrast–Depth Dynamics

**Figure 3:**
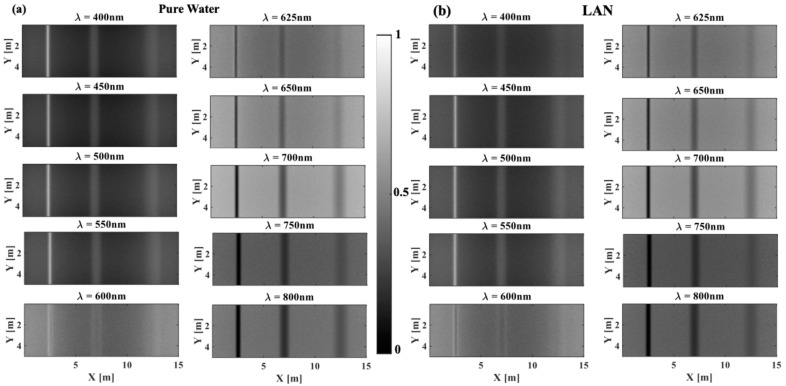

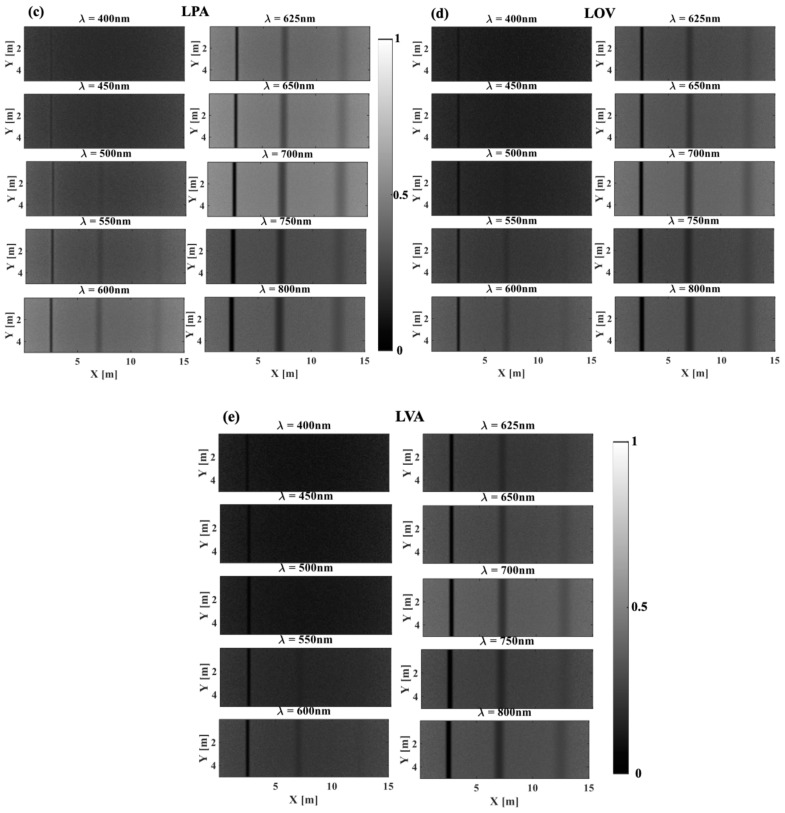
Spectral images of woody debris at different depths in (**a**) pure (clear) water, (**b**) lake Äntu Sinijärv (LAN), (**c**) lake Päijänne (LPA), (**d**) lake Vôrtsjärv (LVO), and (**e**) lake Valkekotinen (LVA) across the 400–800 nm region of the EM spectrum. In this figure, the concentration of chlorophyll in water is *C* = 10 mg.m^−3^. These images were normalized to maximum intensity values in each image.

**Figure 4:**
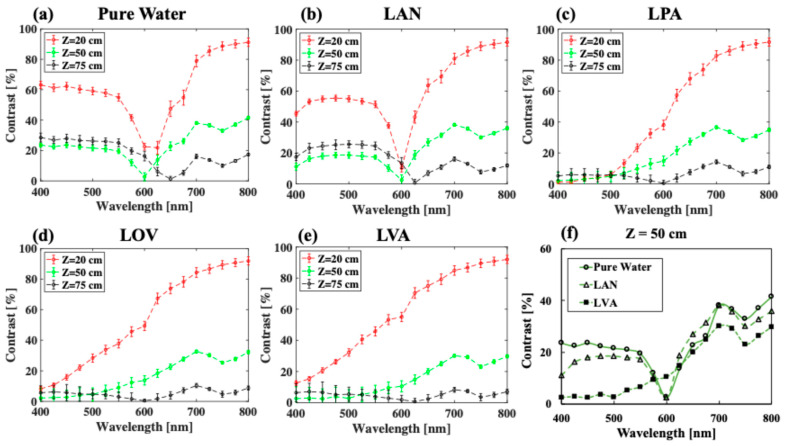
Calculated image contrast of woody debris at different depths across the 400–800 nm region of the EM spectrum considering a concentration of chlorophyll of *C* = 10 mg.m^−3^ in (**a**) pure water, (**b**) LAN, (**c**) LPA, (**d**) LVO, and (**e**) LVA. Figure (**f**) plots the contrast spectra for pure water, LAN, and LVA at a depth of 5.0 m.

Figure 3 shows the spectral images in the wavelength region of 400–800 nm for selected lakes in the order of increasing concentration of phytoplankton pigment, from pure water (Figure 3a) to LVA (Figure 3e). Here, a constant concentration of chlorophyll (scatterer, *C* = 10 mg.m^−3^) is used. Correspondingly, Figure 4 shows the quantitative image contrast spectra at all three depths. In general, contrast decreases with depth, and near infrared (NIR) wavelengths (700–800 nm) produce the best image contrast for intermediate to high concentrations of phytoplankton (LPA, LOV, LVA in Figure 3c–e and Figure 4c–e), whereas shorter wavelengths (400–600 nm) have a slight advantage for zero to low concentrations of phytoplankton (pure water, LAN in Figure 3a,b and Figure 4a,b). For example, considering LVA (with highest concentration of phytoplankton), 800 nm produces a 9× better image contrast than 400 nm for shallow imaging (depth = 2.5 m) and 7.5× for intermediate depth imaging (depth = 5 m). These numbers are roughly 1.5× and 1.8× in pure water (with zero concentration of phytoplankton).

Most interestingly, in pure water and LAN, shorter wavelengths produce high reflected intensity for woody debris (especially at a depth of 25 cm) and low reflected intensity for the background (Figure 3a,b). This is due to the combination effect of low *μ*_a_ values of the background (Figure 2a) and dominant *μ_s_*’ values of woody debris (Figure 2d). There are also notable drops in contrast at wavelengths of 600–650 nm, especially at low concentrations of phytoplankton (Figure 3a,b and Figure 4a,b) and at 750 nm. This is due to the absorption peaks of water in these wavelength regions (Figure 2a).

To provide a better demonstration of the effect of phytoplankton concentration (Figure 2a) on image contrast, Figure 4f compares the contrast spectra for all three water bodies, including pure water (no phytoplankton), lake Äntu Sinijärv (low phytoplankton concentration), and lake Valkekotinen Sinijärv (high phytoplankton concentration), considering a depth of 5 m. Overall, increasing of phytoplankton concentration (increasing of absorption) decreases contrast at most wavelengths except at λ = 600 nm, where a water absorption peak is located. More specifically, at λ = 800 nm, contrast for a depth of 5 m decreases approximately 17%, while *μ*_a_ increases 36% from lake Äntu Sinijärv (*μ*_a_ = 0.028 cm^−1^) to lake Valkekotinen (*μ*_a_ = 0.038 cm^−1^).

### 3.2. The Effect of Chlorophyll Scattering on the Spectral Contrast–Depth Dynamics

**Figure 5:**
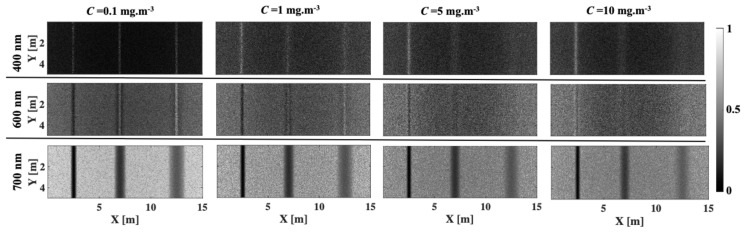
Images of woody debris at three different depths in lake Äntu Sinijärv as scattering increases (increasing concentration of chlorophyll). Images at three wavelengths, 400, 600, and 700 nm, were selected for demonstration.

**Figure 6:**
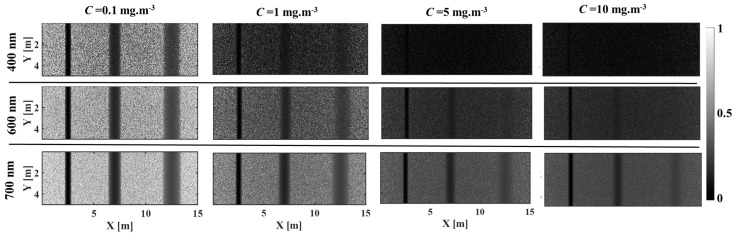
Images of woody debris at three different depths in lake Valkekotinen as scattering increases (increasing concentration of chlorophyll). Images at three wavelengths, 400, 600, and 700 nm, were selected for demonstration.

**Figure 7:**
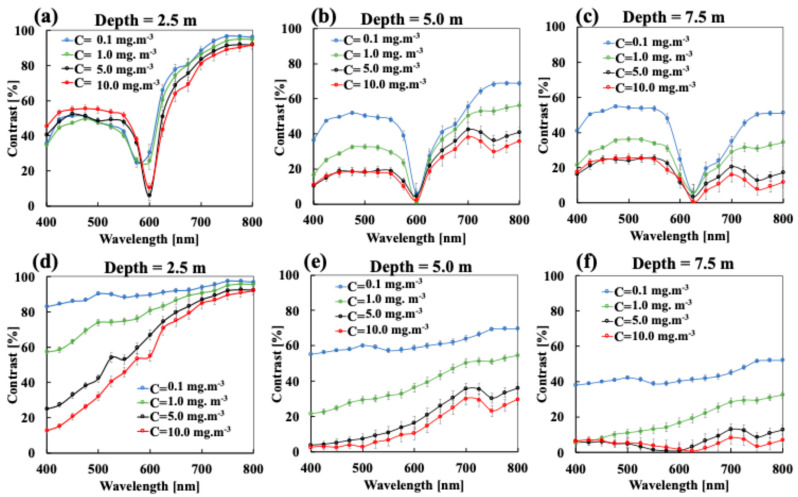
The effect of scattering (concentration of chlorophyll, *C*) on the contrast spectrum in pure water (**a**–**c**) and lake Valkekotinen (**d**–**f**) at different depths: (**a**,**d**) 25 cm, (**b**,**e**) 50 cm, (**c**,**f**) 75 cm.

Figure 5 and Figure 6 show the images at wavelengths of 400, 600, and 700 nm as a function of chlorophyll concentration for lake Äntu Sinijärv and lake Valkekotinen, respectively. Figure 7 quantitatively computes image contrast spectra within wavelengths of 400–800 nm as a function of chlorophyll concentration at all three depths. In general, increasing chlorophyll concentration (Equation (4)) decreases image contrast, especially for lake Valkekotinen with its high phytoplankton concentration (Figure 6 and Figure 7d–f), and as above, contrast decreases with depth. Meanwhile, NIR wavelengths yield mostly better image contrasts than shorter wavelengths. This is because *μ_s_*’ values of chlorophyll are lower at longer wavelengths (Figure 2b). Also, considering lake Valkekotinen with its shallow depth (Z = 2.5 m), contrast at short wavelengths (400–550 nm) is affected by chlorophyll concentration more significantly than that at NIR wavelengths, reduced by 85% at 400 nm and by only 5% at 800 nm when *C* increases from 0.1 to 10 mg.m^−3^ (Figure 6 and Figure 7d). On the other hand, the lake Äntu Sinijärv spectral contrast is strongly distorted at 600 nm, where the water absorption peak is located.

## 4. Discussion and Conclusions

If we apply a contrast cut-off at 10% commonly used in digital imaging [72], i.e., a level below which woody debris can no longer be visualized, a recommendation for satellite photon wavelengths can be made to reach the deepest regions of a lake water body at extreme conditions (lowest or highest concentrations of chlorophyll and phytoplankton).

Considering *C* = 10 mg.m^−3^ (highest concentration of chlorophyll), wavelengths of 700 nm and 800 nm produce sufficient image contrast to distinguish lake bottoms full of woody debris from background media when the depth is within 7.5 m. The reported maximum depth during the rainy season for lake Äntu Sinijärv (low phytoplankton concentration), lake Vôrtsjärv (intermediate phytoplankton concentration), and lake Valkekotinen (highest phytoplankton concentration) is 7.3, 6.0, and 6.0, respectively [54,55,73]. The mean depth over the entire year of these lakes is within 2.5–3.5 m [54,55,73]. Therefore, wavelength of either 700 nm or 800 nm will be able to reach these lakes bottoms and produce sufficient image contrast for visualization in any season (Figure 4). On the other hand, to reveal even more details of lake Äntu Sinijärv at an intermediate depth, wavelengths of 400–550 nm are better options (Figure 4b). In water with low and intermediate phytoplankton concentration, it is advisable to avoid wavelengths of 600–700 nm, where woody debris contrast is strongly distorted by water absorption (Figure 4b,c).

Considering *C* = 0.1 mg.m^−3^ (lowest concentrations of chlorophyll), any wavelength within 400–800 nm can produce sufficient image contrast for lake Valkekotinen, where a minimum contrast of roughly 40% is achievable at a wavelength of 400 nm for a depth of 7.5 m (Figure 6 and Figure 7f). On the other hand, it is advisable to avoid wavelengths of 600–700 nm for viewing the bottom of lake Äntu Sinijärv (Figure 7a–c), where woody debris contrast is strongly distorted by water absorption.

To accurately monitor the water volume of boreal lakes in remote locations, accurate estimation of lake depth from satellite images is necessary. On the other hand, concentrations of phytoplankton and chlorophyll strongly affect the optical penetration depth, while lake depth strongly fluctuates during the year. In this study, we presented a Monte Carlo-based model for boreal lakes and applied it to quantitively establish the contrast–depth dynamic relationship at common satellite wavelengths for earth observation in the Finland study region. The innovative value of this paper is as follows: (a) it highlights the significance of wavelength selection for optimal visualization of boreal lake bottoms, and (b) it shows that image contrast values can be used to estimate lake depth, but with careful treatment of background attenuation. The results show that while near infrared can yield reasonable contrast of woody debris at a depth of 7.5 m in any condition, short wavelengths (400–550 nm) can improve image details at shallow depths (up to 5 m during the drought season) when boral lakes are low in both chlorophyll and phytoplankton. In future work, a database will be trained to automatically cross-reference phytoplankton and chlorophyll concentration (based on dry or wet seasons) and extrapolate depth from an image contrast value. It is worth noting that the results in this study were observed under known optical properties of boreal lake water and of woody debris and do not consider the climate, soil, permafrost, and other aqueous conditions.

## Data Availability

The data are available upon reasonable request.

## References

[1] Riordan B., Verbyla D., McGuire A.D. (2006). Shrinking ponds in subarctic Alaska based on 1950–2002 remotely sensed images. J. Geophys. Res. Biogeosci..

[2] Roach J.K., Griffith B., Verbyla D. (2013). Landscape influences on climate–related lake shrinkage at high latitudes. Global Change Biol..

[3] Carroll M.L., Townshend J., DiMiceli C., Loboda T., Sohlberg R. (2011). Shrinking lakes of the Arctic: Spatial relationships and trajectory of change. Geophys. Res. Lett..

[4] Klein E., Berg E.E., Dial R. (2005). Wetland drying and succession across the Kenai Peninsula lowlands, south-central Alaska. Can. J. For. Res..

[5] Smith L.C., Sheng Y., MacDonald G., Hinzman L. (2005). Disappearing Arctic lakes. Science.

[6] Arnott S.E., Keller B., Dillon P.J., Yan N., Paterson M., Findlay D. (2003). Using temporal coherence to determine the response to climate change in boreal shield lakes. Environ. Monit. Assess..

[7] Hinzman L.D., Bettez N.D., Bolton W.R., Chapin F.S., Dyurgerov M.B., Fastie C.L., Griffith B., Hollister R.D., Hope A., Huntington H.P. (2005). Evidence and implications of recent climate change in northern Alaska and other Arctic regions. Clim. Change.

[8] Kaufman D.S., Schneider D.P., McKay N.P., Ammann C.M., Bradley R.S., Briffa K.R., Miller G.H., Otto-Bliesner B.L., Overpeck J.T., Vinther B.M. (2009). Recent warming reverses long-term Arctic cooling. Science.

[9] Ruckstuhl K.E., Johnson E.A., Miyanishi K. (2008). Introduction. The boreal forest and global change. Philos. Trans. R. Soc. B Biol. Sci..

[10] Baulch H., Schindler D., Turner M., Findlay D., Paterson M., Vinebrooke R. (2005). Effects of warming on benthic communities in a boreal lake: Implications of climate change. Limnol. Oceanogr..

[11] Guzzo M.M., Blanchfield P.J. (2017). Climate change alters the quantity and phenology of habitat for lake trout (Salvelinus na- maycush) in small boreal shield lakes. Can. J. Fish. Aquat. Sci..

[12] Schindler D.W., Bayley S.E., Parker B.R., Beaty K.G., Cruikshank D.R., Fee E.J., Schindler E.U., Stainton M.P. (1996). The effects of climatic warming on the properties of boreal lakes and streams at the Experimental Lakes Area, Northwestern Ontario. Limnol. Oceanogr..

[13] Findlay D., Kasian S., Stainton M., Beaty K., Lyng M. (2001). Climatic influences on algal populations of boreal forest lakes in the Experimental Lakes Area. Limnol. Oceanogr..

[14] Woolway R.I., Kraemer B.M., Lenters J.D., Merchant C.J., O’Reilly C.M., Sharma S. (2020). Global lake responses to climate change. Nat. Rev. Earth Environ..

[15] Choulga M., Kourzeneva E., Zakharova E., Doganovsky A. (2014). Estimation of the mean depth of boreal lakes for use in numerical weather prediction and climate modelling. Tellus A: Dyn. Meteorol. Oceanogr..

[16] Lehner B., Anand M., Fluet-Chouinard E., Tan F., Aires F., Allen G.H., Bousquet P., Canadell J.G., Davidson N., Finlayson C.M. (2024). Mapping the world’s inland surface waters: An update to the global lakes and wetlands database (GLWD v2). Earth Syst. Sci. Data Discuss..

[17] Lehner B., Döll P. (2004). Development and validation of a global database of lakes, reservoirs and wetlands. J. Hydrol..

[18] Messager M.L., Lehner B., Grill G., Nedeva I., Schmitt O. (2016). Estimating the volume and age of water stored in global lakes using a geostatistical approach. Nat. Commun..

[19] Verpoorter C., Kutser T., Seekell D.A., Tranvik L.J. (2014). A global inventory of lakes based on high-resolution satellite imagery. Geophys. Res. Lett..

[20] Arst H., Erm A.J., Herlevi A., Kutser T., Leppäranta M., Reinart A., Virta J. (2008). Optical properties of boreal lake waters in Finland and Estonia. BER.

[21] Ripoll J., Nieto-Vesperinas M., Arridge S.R., Dehghani H. (2000). Boundary conditions for light propagation in diffusive media with nonscattering regions. J. Opt. Soc. Am. A.

[22] Arridge S.R., Dehghani H., Schweiger M., Okada E. (2000). The finite element model for the propagation of light in scattering media: A direct method for domains with nonscattering regions. Med. Phys..

[23] Martelli F., Contini D., Taddeucci A., Zaccanti G. (1997). Photon migration through a turbid slab described by a model based on diffusion approximation. II. Comparison with Monte Carlo results. Appl. Opt..

[24] Hielscher A.H., Alcouffe R.E., Barbour R.L. (1998). Comparison of finite-difference transport and diffusion calculations for photon migration in homogeneous and heterogeneous tissues. Phys. Med. Biol..

[25] Custo A., Wells W.M., Barnett A.H., Hillman E.M., Boas D.A. (2006). Effective scattering coefficient of the cerebral spinal fluid in adult head models for diffuse optical imaging. Appl. Opt..

[26] Ma G., Delorme J.-F., Gallant P., Boas D.A. (2007). Comparison of simplified Monte Carlo simulation and diffusion approximation for the fluorescence signal from phantoms with typical mouse tissue optical properties. Appl. Opt..

[27] Martinelli M., Gardner A., Cuccia D., Hayakawa C., Spanier J., Venugopalan V. (2011). Analysis of single Monte Carlo methods for prediction of reflectance from turbid media. Opt. Express.

[28] Ren X., Chen H., Wang X., Qu G., Wang J., Liang J., Tian J. (2013). Molecular optical simulation environment (MOSE): A platform for the simulation of light propagation in turbid media. PLoS ONE.

[29] Shen H., Wang G. (2010). A tetrahedron-based inhomogeneous Monte Carlo optical simulator. Phys. Med. Biol..

[30] Wang L., Jacques S.L., Zheng L. (1995). MCML—Monte Carlo modeling of light transport in multi-layered tissues. Comput. Methods Programs Biomed..

[31] Wilson B.C., Adam G. (1983). A Monte Carlo model for the absorption and flux distributions of light in tissue. Med. Phys..

[32] Arridge S., Schweiger M., Hiraoka M., Delpy D. (1993). A finite element approach for modeling photon transport in tissue. Med. Phys..

[33] Ajmal A., Boonya-Ananta T., Rodriguez A.J., Le V.D., Ramella-Roman J.C. (2021). Monte Carlo analysis of optical heart rate sensors in commercial wearables: The effect of skin tone and obesity on the photoplethysmography (PPG) signal. Biomed. Opt. Express.

[34] Le V.D., Wang Q., Gould T., Ramella-Roman J.C., Pfefer T.J. (2014). Vascular contrast in narrow-band and white light imaging. Appl. Opt..

[35] Le V.N., Srinivasan V.J. (2020). Beyond diffuse correlations: Deciphering random flow in time-of-flight resolved light dynamics. Opt. Express.

[36] Le V.N., Wang Q., Ramella-Roman J.C., Pfefer T.J. (2012). Monte Carlo modeling of light-tissue interactions in narrow band imag- ing. J. Biomed. Opt..

[37] Wang Q., Le V.N., Ramella-Roman J., Pfefer J. (2012). Broadband ultraviolet-visible optical property measurement in layered turbid media. Biomed. Opt. Express.

[38] Zhu C., Liu Q. (2013). Review of Monte Carlo modeling of light transport in tissues. J. Biomed. Opt..

[39] Fang Q., Boas D.A. (2009). Monte Carlo simulation of photon migration in 3D turbid media accelerated by graphics processing units. Opt. Express.

[40] Yan S., Fang Q. (2020). Hybrid mesh and voxel-based Monte Carlo algorithm for accurate and efficient photon transport modeling in complex bio-tissues. Biomed. Opt. Express.

[41] Yu L., Nina-Paravecino F., Kaeli D., Fang Q. (2018). Scalable and massively parallel Monte Carlo photon transport simulations for heterogeneous computing platforms. J. Biomed. Opt..

[42] Yuan Y., Yu L., Doğan Z., Fang Q. (2018). Graphics processing units-accelerated adaptive nonlocal means filter for denoising three- dimensional Monte Carlo photon transport simulations. J. Biomed. Opt..

[43] Fang Q., Yan S. (2019). Graphics processing unit-accelerated mesh-based Monte Carlo photon transport simulations. J. Biomed. Opt..

[44] Shin H., Jeong S., Lee J.-H., Sun W., Choi N., Cho I.-J. (2021). 3D high-density microelectrode array with optical stimulation and drug delivery for investigating neural circuit dynamics. Nat. Commun..

[45] Takahashi T., Takikawa Y., Kawagoe R., Shibuya S., Iwano T., Kitazawa S. (2011). Influence of skin blood flow on near-infrared spectroscopy signals measured on the forehead during a verbal fluency task. Neuroimage.

[46] Vasung L., Turk E.A., Ferradal S.L., Sutin J., Stout J.N., Ahtam B., Lin P.-Y., Grant P.E. (2019). Exploring early human brain development with structural and physiological neuroimaging. Neuroimage.

[47] Shin H., Son Y., Chae U., Kim J., Choi N., Lee H.J., Woo J., Cho Y., Yang S.H., Lee C.J. (2019). Multifunctional multi-shank neural probe for investigating and modulating long-range neural circuits in vivo. Nat. Commun..

[48] Saloranta T.M., Andersen T. (2007). MyLake—A multi-year lake simulation model code suitable for uncertainty and sensitivity anal- ysis simulations. Ecol. Model..

[49] Vachon D., Lapierre J.F., Del Giorgio P.A. (2016). Seasonality of photochemical dissolved organic carbon mineralization and its relative contribution to pelagic CO_2_ production in northern lakes. J. Geophys. Res. Biogeosci..

[50] Grosbois G., Del Giorgio P.A., Rautio M. (2017). Zooplankton allochthony is spatially heterogeneous in a boreal lake. Freshwater Biol..

[51] Hastie A., Lauerwald R., Weyhenmeyer G., Sobek S., Verpoorter C., Regnier P. (2018). CO_2_ evasion from boreal lakes: Revised estimate, drivers of spatial variability, and future projections. Global Change Biol..

[52] Wolf R., Thrane J.-E., Hessen D.O., Andersen T. (2018). Modelling ROS formation in boreal lakes from interactions between dis- solved organic matter and absorbed solar photon flux. Water Res..

[53] Allesson L., Koehler B., Thrane J.E., Andersen T., Hessen D.O. (2021). The role of photomineralization for CO_2_ emissions in boreal lakes along a gradient of dissolved organic matter. Limnol. Oceanogr..

[54] Street-Perrott F.A., Holmes J.A., Robertson I., Ficken K.J., Koff T., Loader N.J., Marshall J.D., Martma T. (2018). The Holocene isotopic record of aquatic cellulose from lake Äntu Sinijärv, Estonia: Influence of changing climate and organic-matter sources. Quat. Sci. Rev..

[55] Tönno I., Kirsi A.-L., Freiberg R., Alliksaar T., Lepane V., Koiv T., Kisand A., Heinsalu A. (2013). Ecosystem changes in large and shallow Vörtsjärv, a lake in Estonia—Evidence from sediment pigments and phosphorus fractions. Boreal Environ. Res..

[56] Karjalainen L., Kuuluvainen T. (2002). Amount and diversity of coarse woody debris within a boreal forest landscape dominated by Pinus sylvestris in Vienansalo Wilderness, Eastern Fennoscandia. Silva Fenn..

[57] Siitonen J. (2001). Forest management, coarse woody debris and saproxylic organisms: Fennoscandian boreal forests as an example. Ecol. Bull..

[58] Sippola A.L., Siitonen J., Kallio R. (1998). Amount and quality of coarse woody debris in natural and managed coniferous forests near the timberline in Finnish Lapland. Scand. J. Forest Res..

[59] Hecht E. (2001). Optics.

[60] Gordon H.R., Morel A. (1983). In-water algorithms. Remote Assessment of Ocean Color for Interpretation of Satellite Visible Imagery: A Review.

[61] Morel A. (1991). Optics of marine particles and marine optics. Particle Analysis in Oceanography.

[62] Hale G.M., Querry M.R. (1973). Optical constants of water in the 200-nm to 200-μm wavelength region. Appl. Opt..

[63] Cuccia D.J., Bevilacqua F., Durkin A.J., Ayers F.R., Tromberg B.J. (2009). Quantitation and mapping of tissue optical properties using modulated imaging. J. Biomed. Opt..

[64] O’Sullivan T.D., Cerussi A.E., Cuccia D.J., Tromberg B.J. (2012). Diffuse optical imaging using spatially and temporally modulated light. J. Biomed. Opt..

[65] Le V.N., Manser M., Gurm S., Wagner B., Hayward J.E., Fang Q. (2019). Calibration of spectral imaging devices with oxygenation-controlled phantoms: Introducing a simple gel-based hemoglobin model. Front. Phys..

[66] Le V.N., Provias J., Murty N., Patterson M.S., Nie Z., Hayward J.E., Farrell T.J., McMillan W., Zhang W., Fang Q. (2017). Dual-modality optical biopsy of glioblastomas multiforme with diffuse reflectance and fluorescence: Ex vivo retrieval of optical prop- erties. J. Biomed. Opt..

[67] Mobley C.D., Gentili B., Gordon H.R., Jin Z., Kattawar G.W., Morel A., Reinersman P., Stamnes K., Stavn R.H. (1993). Compar- ison of numerical models for computing underwater light fields. Appl. Opt..

[68] Beechie T.J., Sibley T.H. (1997). Relationships between channel characteristics, woody debris, and fish habitat in northwestern Wash- ington streams. Trans. Am. Fish. Soc..

[69] Bilby R.E., Bisson P.A. (1998). Function and distribution of large woody debris. River Ecol. Manag..

[70] Ehrman T.P., Lamberti G.A. (1992). Hydraulic and particulate matter retention in a 3rd-order Indiana stream. J. N. Am. Benthol. Soc..

[71] D’Andrea C., Farina A., Comelli D., Pifferi A., Taroni P., Valentini G., Cubeddu R., Zoia L., Orlandi M., Kienle A. (2008). Time-resolved optical spectroscopy of wood. Appl. Spectrosc..

[72] Allisy-Roberts P.J., Williams J. (2007). Farr’s Physics for Medical Imaging.

[73] Hari P., Pumpanen J., Huotari J., Kolari P., Grace J., Vesala T., Ojala A. (2008). High-frequency measurements of productivity of planktonic algae using rugged nondispersive infrared carbon dioxide probes. Limnol. Oceanogr. Methods.

